# Eco-epidemiological analysis of *Rickettsia parkeri* in domestic dogs and *Amblyomma ovale* ticks in the Atlantic rainforest of Northeast Brazil

**DOI:** 10.1590/S1984-29612024077

**Published:** 2024-12-16

**Authors:** Michellin Pereira de Albuquerque, Mauricio Claudio Horta, Daniele Rosa Xavier de Melo, Gabriela Akemi Cardoso Gagliardi Takeda, Ana Isabel Arraes-Santos, Thiago Fernandes Martins, Adriano Pinter

**Affiliations:** 1 Faculdade de Saúde Pública, Universidade de São Paulo – USP, São Paulo, SP, Brasil; 2 Universidade Federal do Vale do São Francisco – UNIVASF, Petrolina, PE, Brasil; 3 Instituto Pasteur, São Paulo, SP, Brasil; 4 Faculdade de Medicina Veterinária e Zootecnia – FMVZ, Universidade de São Paulo – USP, São Paulo, SP, Brasil

**Keywords:** Eco-epidemiology, public health, serosurvey, spotted fever, tick-borne diseases, Eco-epidemiologia, saúde pública, levantamento sorológico, febre maculosa, doenças transmitida por carrapatos

## Abstract

In Brazil, spotted fever (SF) is caused by two species of *Rickettsia*, both of which are transmitted by *Amblyomma* ticks: *Rickettsia rickettsii*, which results in severe and often fatal cases, and *Rickettsia parkeri,* which causes a mild illness. This study focused on *R. parkeri* in *Amblyomma ovale* ticks from the Maciço de Baturité region, Ceará, Northeast Brazil, an area endemic for SF with mild symptoms. We examined 60 domestic dogs with access to the forest for ticks and *Rickettsia* seroprevalence. A landscape analysis was conducted in all forest patches within 2–10 km from the main forest edge. In total, 125 *A. ovale* ticks were collected from 30 dogs (50%). DNA from 65 ticks was tested using genus-specific *Rickettsia* primers. Three (4.6%) tick specimens tested positive for *R. parkeri* while the *Rickettsia* seroprevalence among the dogs was 55% (33/60). A probable occurrence of *Rickettsia* transmission was observed in the fragmented Atlantic rainforest, which has 1,019 ha of preserved land and 50.6 km of perimeter border. The land's characteristics allow for semi-domiciled dogs to access forest fragments, where *A. ovale* ticks are commonly present. Infected ticks may parasitize the dogs, which then transport the ticks into homes, potentially transmitting SF-causing bacteria to humans.

## Resumo

No Brasil, a febre maculosa (FM) é causada por duas espécies de *Rickettsia*, ambas transmitidas por carrapatos *Amblyomma: Rickettsia rickettsii*, ocasionando casos graves e frequentemente fatais, e *Rickettsia parkeri*, que causa uma doença leve. Este estudo concentrou-se em *R. parkeri* em carrapatos *Amblyomma ovale* na região do Maciço de Baturité, Ceará, Nordeste do Brasil, área endêmica para FM com sintomas leves. Foram examinados 60 cães domésticos com acesso à floresta para carrapatos e soroprevalência de *Rickettsia*. Conduziu-se uma análise da paisagem em todos os fragmentos florestais dentro de 2–10 km da borda principal da floresta. No total, 125 carrapatos *A. ovale* foram coletados de 30 cães (50%). Testou-se o DNA de 65 carrapatos, usando-se *primers* de *Rickettsia* gênero-específico. Três (4,6%) espécimes de carrapatos testaram positivo para *R. parkeri*, enquanto a soroprevalência de *Rickettsia* entre os cães foi de 55% (33/60). Observou-se uma provável ocorrência de transmissão de *Rickettsia* na fragmentada Mata Atlântica, que possui 1.019 ha de terra preservada e 50,6 km de borda perimetral. As características da área permitem que cães semidomiciliados acessem fragmentos florestais, onde carrapatos *A. ovale* são comumente presentes. Carrapatos infectados podem parasitar cães, que então os transportam para as casas, potencialmente transmitindo bactérias causadoras da FM aos humanos.

## Introduction

Spotted fever group (SFG) rickettsiae, the causal agents of mild-to-severe spotted fever (SF) in humans, are primarily transmitted by ticks. The bacterium *Rickettsia parkeri* is known to cause milder tick-borne SF in humans than the highly pathogenic *Rickettsia rickettsii* (Faccini-Martínez et al., 2018). In general, the epidemiology of the disease variety is directly determined by species of *Rickettsia*, as well as that of its tick vector (Szabó et al., 2013).

In Brazil, the *R. parkeri* strain Atlantic rainforest has been detected in several tick species; however, *Amblyomma ovale* has been demonstrated to be the main vector of this bioagent among humans (Faccini-Martínez et al., 2018; Szabó et al., 2013). This tick species has a broad geographical distribution throughout Brazil, including the Amazon region, Atlantic rainforest, floodplains, and savannas (Bitencourth et al., 2019). Adult ticks primarily feed on carnivores, while subadults feed on small rodents (Szabó et al., 2013). In anthropogenic environments, a specific cycle has been described in which adult *A. ovale* ticks primarily feed on domesticated dogs with unrestricted access to forest areas, posing a potential threat once these dogs carry infected ticks from the forest into their home, where ticks can be accidentally transferred to humans (Fournier et al., 2019).

Human cases of *R. parkeri* infection have been reported in several areas throughout Brazil, including the Maciço de Baturité region in Ceará (CE), Northeast Brazil (Brasil, 2024; Moerbeck et al., 2016). This area, located between the central semiarid backlands and the coastal metropolitan region of Fortaleza, is naturally isolated within the Atlantic rainforest. Even though Maciço de Baturité is in a semiarid region of a mountain chain, having an average altitude of 800 m allows the area to maintain a moist and mild climate, lower temperatures, higher precipitation index, and increased biodiversity. Previous studies have classified this region as endemic for SF; however, they reported only mild human cases, stemming from *Rhipicephalus linnaei* (previously identified as *Rhipicephalus sanguineus* senso lato) and *A. ovale* ticks carrying *R. parkeri* strain Atlantic rainforest (Moerbeck et al., 2016). Therefore, the objective of this study was to investigate the occurrence of *R. parkeri* in *A. ovale* ticks, assess seroprevalence in dogs in areas where human cases of SF disease have been reported, and evaluate for any correlation with the landscape of the Maciço de Baturité region.

## Materials and Methods

### Study area and sampling

Between May 24 and 26, 2015, the Health Department of the Municipality of Guaramiranga conducted a field investigation of four cases of SF in humans in the Maciço de Baturité region. All four cases occurred in 2013 and were laboratory confirmed by the Brazilian Ministry of Health. To evaluate the risk of SF in this area, a cross-sectional study was conducted on a cohort of semi-domiciled dogs residing in households near those where mild cases of SF had been confirmed.

A total of 60 dogs were selected using on the following criteria: raised unrestrained, free access to forest areas, resided without leaving the area, and clinically healthy (Pinter et al., 2008). During sampling, each dog owner responded to a previously validated epidemiological questionnaire addressing the breed, age, and sex of their dogs. Additionally, the geographical coordinates for each dog dwelling were obtained using the Global Positioning System (GPS; Garmin ® E-Trex-30, Olathe, KA, USA).

### Serum collection and immunofluorescence assay (IFA)

A 1 mL sample of blood was obtained from each dog through a puncture in the cephalic vein of one of the front legs. The samples were stored in sterile tubes marked with the dog’s identification and transported to the laboratory, where they were centrifuged at 3,000 g for 5 min to separate the serum aliquots, which were transferred to another 2 mL microtube and stored at -20 °C until processing by IFA. All 60 serum samples were submitted for IFA in the Laboratory of Seroepidemiology of the Superintendência de Controle de Endemias in São Paulo, following the protocol described by Horta et al. (2004). IFA was performed twice using antigens against Vero cells infected with *R. rickettsii* strain Taiaçu (Pinter & Labruna, 2006) or *R. parkeri* strain AT24 (Silveira et al., 2007), fixed on microscope slides for immunofluorescence. Each slide was stained with fluorescein isothiocyanate and visualized under a fluorescence microscope at 400× magnification. Only samples with an IFA reaction at a dilution ≥ 1:64 were considered positive and submitted for further testing against the higher no-reagent dilution. In samples that showed endpoint titers for *R. parkeri* that were ≥ four-fold higher than those for *R. rickettsii*, it was considered that *R. parkeri* was the probable homologous antigen (PHA) or a very closely related genotype, as described by Oliveira et al. (2019) and Horta et al. (2004).

### Collection of ticks and taxonomy

Each dog was searched for ticks for five min, with any ticks removed using tweezers and placed in tubes. Adult ticks were morphologically identified using a stereomicroscope, as described by Barros-Battesti et al. (2006) and Šlapeta et al. (2022). Ticks were kept at room temperature for 3 days until they were transported to the laboratory, where live ticks were stored in a -80 °C ultra-low temperature freezer and dead ticks were stored in alcohol for further deposit in a zoological collection.

### Molecular and phylogenetic analyses

Each adult *A. ovale* tick was taken from the -80 °C freezer and placed over a cold platform device at -20 °C, where the legs were removed using a single sterile scalpel. The legs were transferred to a microcentrifuge tube containing 50 µL TE buffer (Tris HCl 10 mM, 1 mM EDTA; pH, 8.0) and heated at 99 °C for 15 min (Pinter & Labruna, 2006) with modifications for deoxyribonucleic acid (DNA) extraction.

The tick samples were tested for the presence of *Rickettsia* spp. by conventional polymerase chain reaction (PCR) using primer sets targeting the *glt*A (primers CS-78, CS-323) (Labruna et al., 2004a), *omp*A (*Rr*190.70p, *Rr*190.602n) (Regnery et al., 1991), and *htr*A (17k-5, 17K-3) (Labruna et al., 2004b) genes using the DreamTaq Green PCR Master Mix (Thermo Fisher Scientific, Waltham, MA, USA) according to the manufacturer’s instructions. Positive (*R. rickettsii*) and negative (autoclaved DNA-free Milli-Q water) controls were included for each reaction. All products were analyzed by electrophoresis using a 2% agarose gel.

Amplicons of the expected size were purified with ExoSAP-IT (Thermo Fisher Scientific, Waltham, MA, USA) and sequenced using the BigDye Terminator version 3.1 Cycle Sequencing Kit and an ABI 3500 Genetic Analyzer (both from Applied Biosystems, Foster City, CA, USA). The sequences were manually edited, generating consensus sequences using ChromasPro 1.5 program (Technelysium, Qld, Australia). The consensus sequences were automatically aligned using the multiple alignment algorithm ClustalW in MEGA X v. 10.1.7 (Kumar et al., 2018). The sequences were compared with those in the GenBank database using Basic Local Alignment Search Tool (BLAST) analysis to determine the similarities between *Rickettsia* spp.

The generated gene fragments were concatenated and submitted to phylogenetic tree models to evaluate their proximity, after which they were compared with sequences available in GenBank. A phylogenetic tree was constructed using maximum likelihood analysis with PHYML (v 3.0) and the General Time Reversible (GTR)+G evolutionary model (Kimura, 1980). Bootstrap values were obtained from 1,000 randomly generated trees. All sequences generated in this study have been deposited to GenBank under accession numbers MW362872, MW362873, MW362874, MW362875, MW362876, MW362877, MW362878, MW362879, and MW362880.

### Statistical analysis

The total number of *A. ovale* ticks collected was used to determine the mean intensity, mean abundance, and whether or not the population fit a negative binomial distribution. Statistical analyses were performed using Quantitative Parasitology 3.0 (Reiczigel et al., 2019), and descriptive statistics were presented using a bar graph created in Microsoft Excel, depicting both absolute numbers (n) and percentages (%).

### Landscape analysis

To characterize the rainforest fragments where the dogs roamed, high-quality satellite images obtained in 2015 were visualized using software and satellite-provided imagery from Google Earth Pro (Google, LLC). The borders of the forest fragments were identified under high levels of magnification, which allowed the measurement of three different landscape variables: perimeter and area of the forest fragments within a 2,000 m radius and connected forest areas within a 10,000 m radius from each dog’s home, according to Scinachi et al. (2017).

## Results

Of the 60 dogs evaluated, 33 (55%) yielded seroreactivity (with titer ≥ 64) against the *R. parkeri* antigen, with titers ranging from 64–16,384, while 40% (24/60) showed seroreactivity against the *R. rickettsii* antigen, with titers ranging from 64–8,192. A total of 27 dogs (45%) tested negative for both antigens (Table 1). Furthermore, 66.7% (22/33) of the seropositive samples were considered homologous to *R. parkeri*; however, for the remaining 11 dogs (33.3%), it was not possible to determine the PHA, since they expressed similar titers for both antigens.

**Table 1:** Identification of dog samples according to age and antibody titers by indirect immunofluorescence assay (IFA) and probable homologous antigen (PHA) for two *Rickettsia* species tested in serum from dogs of Maciço de Baturité, Ceará, Brazil, 2015.

**Sample**	**Age**	**IFA titers for the following antigens**		**AT**	**PHA**
		***Rickettsia parkeri***	***Rickettsia rickettsii***		
CE1	2 y	64	Negative	Het	Undetermined
CE2	4 y	128	Negative	Hom	*Rickettsia parkeri*
CE3	7 y	2,048	1,024	Het	Undetermined
CE4	8 y	2,048	2,048	Het	Undetermined
CE5	9 y	2,048	512	Hom	*Rickettsia parkeri*
CE6	4 y	1,024	512	Het	Undetermined
CE9	1 y	1,024	1,024	Het	Undetermined
CE12	8 y	2,048	512	Hom	*Rickettsia parkeri*
CE13	2 y	16,384	8,192	Het	Undetermined
CE14	3 y	2,048	512	Hom	*Rickettsia parkeri*
CE17	2 y	2,048	1,024	Het	Undetermined
CE18	8 m	4,096	512	Hom	*Rickettsia parkeri*
CE21	2 y	2,048	512	Hom	*Rickettsia parkeri*
CE22	10 y	128	Negative	Hom	*Rickettsia parkeri*
CE23	1 y	1,024	256	Hom	*Rickettsia parkeri*
CE24	2 y	256	64	Hom	*Rickettsia parkeri*
CE25	3 y	256	Negative	Hom	*Rickettsia parkeri*
CE26	2.5 y	128	Negative	Hom	*Rickettsia parkeri*
CE27	1 y	512	256	Het	Undetermined
CE28	1.5 y	16,384	4,096	Hom	*Rickettsia parkeri*
CE34	1 y	1,024	256	Hom	*Rickettsia parkeri*
CE35	4 y	1,024	256	Hom	*Rickettsia parkeri*
CE36	2.5 y	512	64	Hom	*Rickettsia parkeri*
CE37	3 y	512	Negative	Hom	*Rickettsia parkeri*
CE40	4 y	256	256	Het	Undetermined
CE42	2 y	1,024	256	Hom	*Rickettsia parkeri*
CE43	7 m	128	64	Het	Undetermined
CE47	8 y	8,192	4,096	Het	Undetermined
CE52	9 y	64	Negative	Het	Undetermined
CE53	4 y	128	Negative	Hom	*Rickettsia parkeri*
CE54	1 y	256	256	Het	Undetermined
CE56	8 y	128	128	Het	Undetermined
CE57	2 y	256	64	Hom	*Rickettsia parkeri*

AT = Antibody titers; Het = Heterologous; Hom = Homologous; y = years; m = months.

The prevalence of dogs infested with *R. linnaei* and/or *A. ovale* ticks was 73.33%, while the prevalence was 50% for *A. ovale* alone. In total, 88 and 125 *R. linnaei* and *A. ovale* ticks were collected, respectively. All *A. ovale* collected ticks were adults, 68 males (M) and 57 females (F), and their prevalence showed the fit of a negative binomial distribution as acceptable (*P*-value from chi-squared test = 0.4598). The mean intensity was 4.17 ticks per dog (24-1, maximum and minimum ticks count) with a 95% confidence interval (CI) of 2.97–6.52, and the mean abundance was 2.08 ticks per dog with a 95% CI of 1.35–3.45 (Table 2).

**Table 2:** Statistical analysis of examined and infested semi-domiciled dogs by *A. ovale* and *R. linnaei* from Maciço de Baturité, Ceará, Brazil, 2015.

**Tick**	**Examined dogs**	**Infested dogs**	**Total of ticks**	**Mean abundance**	**Mean intensity**	**Prevalence**
***Amblyomma ovale***	60	30	125	2.08	4.16	50,0%
***Rhipicephalus linnaei***	60	26	88	1.46	3.38	43.3%

**Figure 1:**
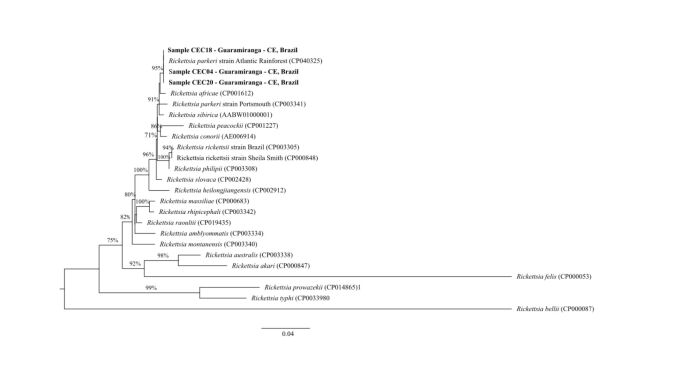
Phylogenetic tree of fragments of the *glt*A, *omp*A, *htr*A gene of *Rickettsia*, detected in *A. ovale* ticks during investigation of Maciço de Baturité region, Ceará State, Northeast Brazil, inferred by maximum likelihood analysis with GTR + G evolutionary model. The figures in the branches indicate values of statistical support (70% “cut-off").

A total of 65 *A. ovale* ticks survived transport to the laboratory and were subjected to genomic DNA extraction. Of these ticks, 4.6% (3/65) yielded positive results for SFG *Rickettsia* using PCR targeting *glt*A, *omp*A, and *htr*A genes. All three samples were successfully sequenced using BLAST analysis, with samples from ticks CEC04 (M), CEC18 (F), and CEC20 (M) yielding100% identity for *glt*A, *omp*A, and *htr*A (350/350, 491/491, and 497/497, respectively) to partial gene sequences of *R. parkeri* strain Atlantic rainforest deposited in GenBank under accession number CP040325. All of the generated sequences had a close phylogenetic relation to the *R. parkeri* strain found in the Atlantic rainforest (Figure 1).

**Figure 2:**
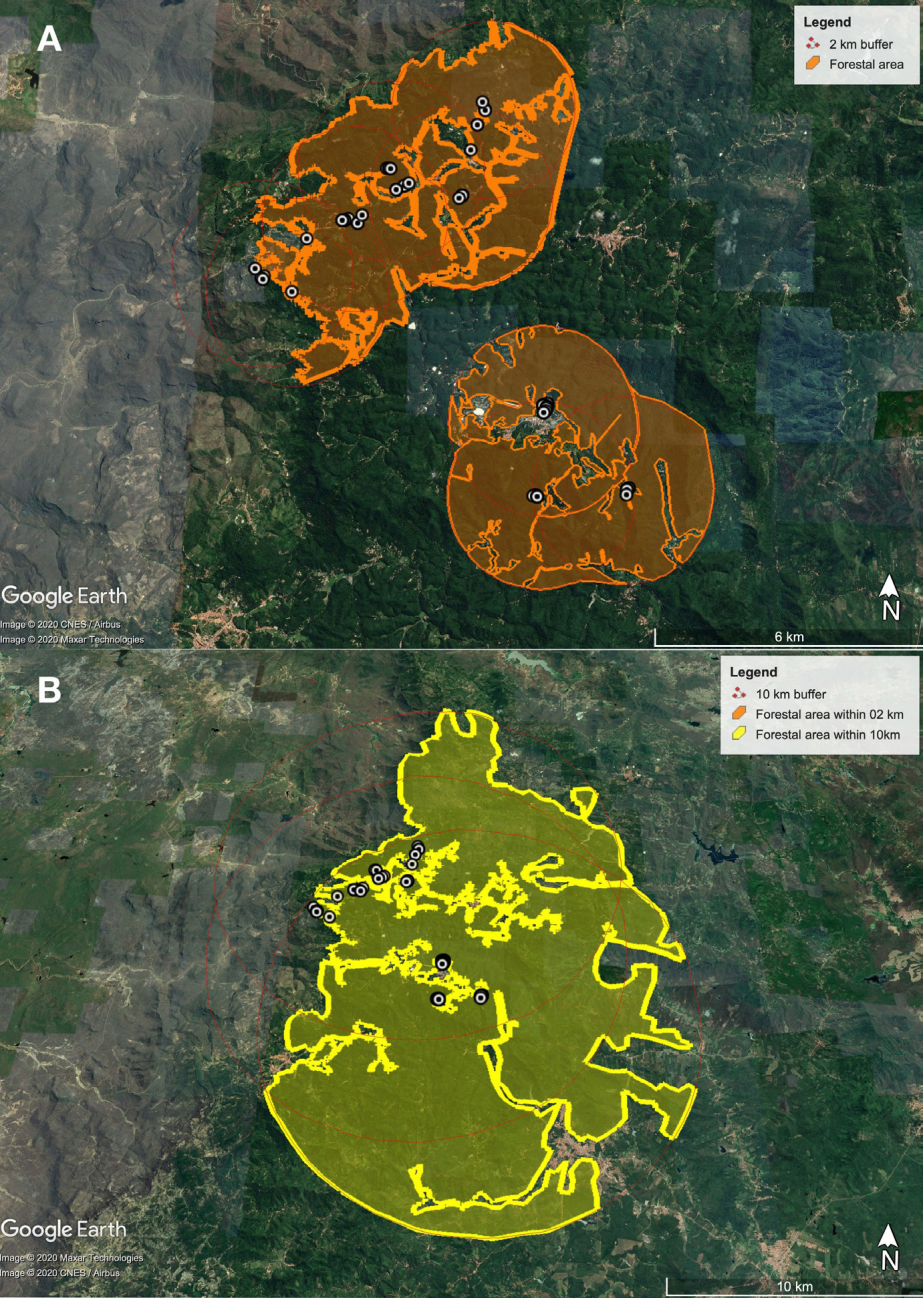
(a) Analysis of forest area where dogs were sampled (white dots) within 2 km radium; (b) Analysis of forest area where dogs were sampled (white dots) within 10 km radium, during investigation of Maciço de Baturité region, Ceará State, Northeast Brazil. Every point on the map has at least one positive dog.

The dogs included in the study cohort roamed in two main forest patches, the north and south patches, composed of naturally preserved Atlantic rainforest remains. All of the dogs were raised unrestrained and were free to roam the forest. The northern patch included 2,631 ha of forest with 104 km of edge perimeter, and the southern patch included 2,130 ha of forest with 94.5 km of edge perimeter. When the analysis buffer was expanded to 10 km, both patches became structurally connected by forest corridors, with a total forest area of 29,732 ha (Figure 2A). Therefore, ratio of the total forest area within a 10 km radius to the total combined forest area within a 2 km radius was 6.24, demonstrating that this region presents a highly preserved forest, with a very high structural connection (Figure 2B).

## Discussion

In the present study, we observed the *R. parkeri* strain Atlantic rainforest in naturally infected *A. ovale* adult ticks collected from semi-domiciled dogs near the most likely infection sites for four human cases of SF in the Maciço de Baturité region of Brazil. *A. ovale* ticks are major carnivore parasites, to which domesticated dogs are exposed when entering forest patches. Dogs, therefore, may play an important role in carrying adult ticks from forests to households, where ticks may then be transferred to human hosts (Suzin et al., 2022; Fournier et al., 2019).

The seroprevalence among the study cohort indicated that the dogs had been exposed to *R. parkeri-*infected ticks. Serological results indicated that ≥ 55% (33/60) of the dogs were exposed to SFG *Rickettsia*. Because of the four-fold titer criteria, it is possible to claim that among the seropositive dogs, 60.6% were most likely to be exposed to *R. parkeri* infection. Furthermore, the majority of the dogs in the cohort were infested by adult *A. ovale* ticks. Our findings on canine seroprevalence suggest significant exposure to SFG rickettsiae, most likely *R. parkeri,* compared to studies performed in southern Brazil. In other *R. parkeri*–endemic areas, Krawczak et al. (2016) and Faccini-Martínez et al. (2020), found seropositivity rates of approximately 20% and 49% of seropositivity in dogs, respectively. Barbieri et al. (2014) and Szabó et al. (2013), however, observed seropositivity rates of approximately 67% and 88%, respectively. Faccini-Martínez et al. (2020) suggested that these high-range seroprevalence values are due to variances in the infection rates of *R. parkeri* in *Amblyomma* spp. ticks, which was corroborated by the results of this study.

Our results also demonstrated a positivity rate of 4.6% for *R. parkeri* infection in *A. ovale* ticks, which is average when compared to the rates of 0.7–14.8% found in other areas (Barbieri et al., 2014; Faccini-Martínez et al., 2020; Krawczak et al., 2016; Szabó et al., 2013). The presence of infected ticks in the study area supports our serological findings in semi-domiciled dogs as well as the characterization of the area as enzootic for *R. parkeri* circulation. It is important to point out that one limitation of this study is that the evaluation of *Rickettsia* spp. infection in *A. ovale* ticks was performed using only the legs of the ticks; therefore, we cannot disregard the possible decrease in sensitivity compared to other studies that tested the whole tick. Moreover, the occurrence of four human SF cases in 2013 confirmed the circulation of *R. parkeri*. Based on our results, the Maciço de Baturité region may be characterized as an area very likely to be the infection site of the previously reported human cases. State Epidemiological Surveillance has reported > 30 confirmed human cases between 2014 and 2022 in municipalities located within the Maciço do Baturité region (Brasil, 2024), reinforcing our conclusions and corroborating other studies of this region which have also observed *R. parkeri* in *A. ovale* ticks (Bitencourth et al., 2019; Moerbeck et al., 2016; Oliveira, 2016; Silva et al., 2017).

To the best of our knowledge, the present study is the first to perform a serosurvey among a dog cohort in this area. Our results showed that dogs play an important role as vectors, and that dogs also play an important role in the disease cycle, acting as transportation for ticks from the forest into houses.

Our analysis of the *glt*A, *omp*A, and *htr*A genes showed 100% identity to the corresponding sequence of *R. parkeri* strain Atlantic rainforest in three samples (CE04, CE18, and CE20). Phylogenetic analysis performed with fragments of *glt*A, *omp*A, and *htr*A genes showed that all of the generated sequences were closely phylogenetically related to the *R. parkeri* strain Atlantic rainforest, in concordance with previous studies (Moerbeck et al., 2016). Other reports of *R. parkeri*-infected *A. ovale* ticks in other regions of the high- and low-altitude Atlantic rainforests in Brazil (Faccini-Martínez et al., 2020; Oliveira et al., 2019; Paixão Sevá et al., 2019; Sabatini et al., 2010) suggest that human cases caused by this pathogen may have gone undiagnosed and underreported (Faccini-Martínez et al., 2018).

The landscape analyses showed that all of the sampled dogs lived in close contact with the edges of forest fragments, and interacted with one of the two main forest fragments in the region. These results differ from what was reported in SF-endemic areas in southeastern Brazil, where the vector is *Amblyomma aureolatum* and the SF agent is *R. rickettsii*. For the *A. aureolatum* area, dogs interacting with forest fragments > 1,500 ha were not fed on by infected ticks, nor did they show seroreactivity to *Rickettsia* spp.; however, a higher seroprevalence in dogs interacting with forest fragments < 850 ha was reported, which may indicate a dilution effect in larger preserved forest fragments (Scinachi et al., 2017).

The larger forest fragments (2,631 h) in the present study did not prevent the circulation of *R. parkeri* among the tick population; therefore, the *R. parkeri* life cycle and infection of dogs are not likely to be dependent on forest fragmentation, which may occur even in large forest areas. In the present study, the total connected forest area within a 10 km radius was > 29,000 ha; however, for the *A. aureolatum*-borne *R. rickettsii* endemic areas in southeastern Brazil, areas with connected forest patches > 14,000 ha did not present seropositive dogs or ticks. These findings may show that for *A. ovale*-borne *R. parkeri*, highly preserved areas and more connected forest patches are not protective factors, as shown by Scinachi et al. (2017).

In the present study, the collected *R. linnaei* ticks were not tested for *Rickettsia* spp., even though previous studies have detected *Rickettsia* genetic material in this species (Silva et al., 2017). It is not possible to exclude that an infection in *R. linnaei* was not accidental; therefore, it would be uninformative for the aim of the present study.

Our results demonstrate the importance of acarological and epidemiological surveillance in the Ceará Atlantic rainforest, where human cases of SF have been reported in recent years. Control measures, such as managing tick infestations in dogs and promoting public awareness campaigns for local residents as well as travelers about the risk of exposure to known transmission areas of SF, must be used as a preventive measure to decrease the number of SF cases in this area.

## Conclusions

Our results confirmed the presence of *R. parkeri* strain Atlantic rainforest in the Maciço de Baturité region of Ceará, Brazil for which *A. ovale* ticks may be the primary vector. The presence of domesticated dogs from houses near the forest with unrestricted access to forest areas is the cause of an *R. parkeri* strain Atlantic rainforest seroprevalence of approximately 50%. Therefore, the role of dogs in the SF cycle in this region should be studied further.

## References

[B001] Barbieri ARM, Moraes J, Nieri-Bastos FA, Souza JC, Szabó MP, Labruna MB (2014). Epidemiology of *Rickettsia* sp. strain Atlantic rainforest in a spotted fever-endemic area of southern Brazil. Ticks Tick Borne Dis.

[B002] Barros-Battesti D, Arzua M, Bechara H (2006). Carrapatos de importância médico-veterinária da região neotropical: um guia ilustrado para identificação de espécies..

[B003] Bitencourth K, Amorim M, Oliveira SV, Voloch CM, Gazêta GS (2019). Genetic diversity, population structure, and rickettsias in *Amblyomma ovale* in areas of epidemiological interest for spotted fever in Brazil. Med Vet Entomol.

[B004] Brasil (2024). Casos confirmados de febre maculosa: Brasil, grandes regiões e unidades federadas (infecção) - 2007 a 2024.

[B005] Faccini-Martínez AA, Oliveira SV, Cerutti C, Labruna MB (2018). Febre maculosa por *Rickettsia parkeri* no Brasil: condutas de vigilância epidemiológica, diagnóstico e tratamento. J Health Biol Sci.

[B006] Faccini-Martínez AA, Muñoz-Leal S, Krawczak FS, Acosta ICL, Martins TF, Serpa MCA (2020). Epidemiological aspects of *Rickettsia parkeri* in the Atlantic forest biome of Espírito Santo state, Brazil. Ticks Tick Borne Dis.

[B007] Fournier GFSR, Pinter A, Santiago R, Muñoz-Leal S, Martins TF, Lopes MG (2019). A high gene flow in populations of *Amblyomma ovale* ticks found in distinct fragments of Brazilian Atlantic Rainforest. Exp Appl Acarol.

[B008] Horta MC, Labruna MB, Sangioni LA, Vianna MCB, Gennari SM, Galvão MAM (2004). Prevalence of antibodies to spotted fever group Rickettsiae in humans and domestic animals in a Brazilian spotted fever-endemic area in the state of São Paulo, Brazil: serologic evidence for infection by *Rickettsia rickettsii* and another spotted fever group Rickettsia. Am J Trop Med Hyg.

[B009] Kimura M (1980). A simple method for estimating evolutionary rates of base substitutions through comparative studies of nucleotide sequences. J Mol Evol.

[B010] Krawczak FS, Muñoz-Leal S, Guztzazky AC, Oliveira SV, Santos FCP, Angerami RN (2016). *Rickettsia* sp. strain Atlantic Rainforest infection in a patient from a spotted fever-endemic area in southern Brazil. Am J Trop Med Hyg.

[B011] Kumar S, Stecher G, Li M, Knyaz C, Tamura K (2018). MEGA X: molecular evolutionary genetics analysis across computing platforms. Mol Biol Evol.

[B012] Labruna MB, Whitworth T, Bouyer DH, McBride J, Camargo LMA, Camargo EP (2004). *Rickettsia bellii* and *Rickettsia amblyommii* in *Amblyomma* ticks from the State of Rondônia, Western Amazon, Brazil. J Med Entomol.

[B013] Labruna MB, Whitworth T, Horta MC, Bouyer DH, McBride JW, Pinter A (2004). *Rickettsia* species infecting *Amblyomma cooperi* ticks from an area in the state of São Paulo, Brazil, where Brazilian spotted fever is endemic. J Clin Microbiol.

[B014] Moerbeck L, Vizzoni VF, Machado-Ferreira E, Cavalcante RC, Oliveira SV, Soares CAG (2016). *Rickettsia* (Rickettsiales: Rickettsiaceae) vector biodiversity in high altitude Atlantic forest fragments within a semiarid climate: a new endemic area of spotted-fever in Brazil. J Med Entomol.

[B015] Oliveira PB, Harvey TV, Fehlberg HF, Rocha JM, Martins TF, da Acosta ICL (2019). Serologic and molecular survey of *Rickettsia* spp. in dogs, horses and ticks from the Atlantic Rainforest of the state of Bahia, Brazil. Exp Appl Acarol.

[B016] Oliveira SV (2016). Tick-borne spotted fever in the northeast of Brazil: the series of cases a new endemic area. Rev Med UFC.

[B017] Paixão Sevá A, Martins TF, Munõz-Leal S, Rodrigues AC, Pinter A, Luz HR (2019). A human case of spotted fever caused by *Rickettsia parkeri* strain Atlantic Rainforest and its association to the tick *Amblyomma ovale.*. Parasit Vectors.

[B018] Pinter A, Horta MC, Pacheco RC, Moraes-Filho J, Labruna MB (2008). Serosurvey of *Rickettsia* spp. in dogs and humans from an endemic area for Brazilian spotted fever in the State of São Paulo, Brazil. Cad Saude Publica.

[B019] Pinter A, Labruna MB (2006). Isolation of *Rickettsia rickettsii* and *Rickettsia bellii* in cell culture from the tick *Amblyomma aureolatum* in Brazil. Ann N Y Acad Sci.

[B020] Regnery RL, Spruill CL, Plikaytis BD (1991). Genotypic identification of rickettsiae and estimation of intraspecies sequence divergence for portions of two rickettsial genes. J Bacteriol.

[B021] Reiczigel J, Marozzi M, Fábián I, Rózsa L (2019). Biostatistics for parasitologists: a primer to quantitative parasitology. Trends Parasitol.

[B022] Sabatini GS, Pinter A, Nieri-Bastos FA, Marcili A, Labruna MB (2010). Survey of ticks (Acari: Ixodidae) and their *Rickettsia* in an Atlantic rain forest reserve in the state of São Paulo, Brazil. J Med Entomol.

[B023] Scinachi CA, Takeda GACG, Mucci LF, Pinter A (2017). Association of the occurrence of Brazilian spotted fever and Atlantic rain forest fragmentation in the São Paulo metropolitan region, Brazil. Acta Trop.

[B024] Silva AB, Duarte MM, da Costa Cavalcante R, Oliveira SV, Vizzoni VF, de Lima Duré AÍ (2017). *Rickettsia rickettsii* infecting *Rhipicephalus sanguineus* sensu lato (Latreille 1806), in high altitude atlantic forest fragments, Ceara State, Brazil. Acta Trop.

[B025] Silveira I, Pacheco RC, Szabó MPJ, Ramos HGC, Labruna MB (2007). *Rickettsia parkeri* in Brazil. Emerg Infect Dis.

[B026] Šlapeta J, Halliday B, Chandra S, Alanazi AD, Abdel-Shafy S (2022). *Rhipicephalus linnaei* (Audouin, 1826) recognised as the “tropical lineage” of the brown dog tick *Rhipicephalus sanguineus* sensu lato: neotype designation, redescription, and establishment of morphological and molecular reference. Ticks Tick Borne Dis.

[B027] Suzin A, Silva MX, Tognolli MH, Vogliotti A, Adami SF, Moraes MFD (2022). Ticks on humans in an Atlantic rainforest preserved ecosystem in Brazil: Species, life stages, attachment sites, and temporal pattern of infestation. Ticks Tick Borne Dis.

[B028] Szabó MPJ, Nieri-Bastos FA, Spolidorio MG, Martins TF, Barbieri AM, Labruna MB (2013). *In vitro* isolation from *Amblyomma ovale* (Acari: Ixodidae) and ecological aspects of the Atlantic Rainforest *Rickettsia*, the causative agent of a novel spotted fever rickettsiosis in Brazil. Parasitology.

